# Supplementary material for: Fatty acid metabolism in aggressive B-cell lymphoma is inhibited by tetraspanin CD37
Source: Nat Commun. 2022 Sep 13;13:5371. doi: 10.1038/s41467-022-33138-7 (PMC9470561; doi:10.1038/s41467-022-33138-7)

Supplementary Fig. 1

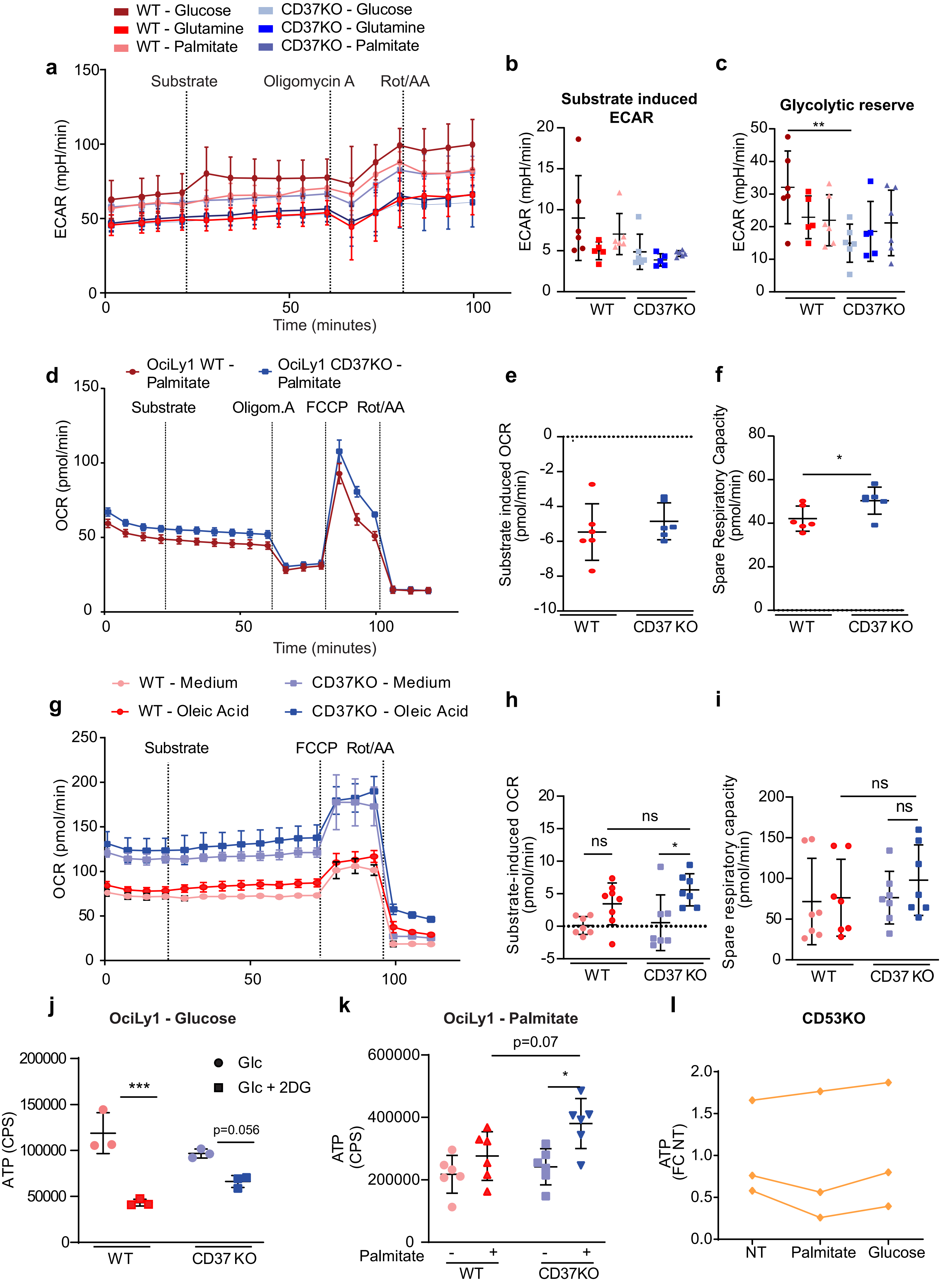

Figure S1. CD37KO lymphoma cells do not respond to glucose or oleic acid in presence of alternative substates.

Seahorse analysis of mitochondrial respiration and glycolysis in human B cell lymphoma (BJAB cells). WT and CD37KO cells were incubated in nutrient-rich medium for 2 hours and subjected to an acute substrate injection of glucose (10 mM) (n=6), glutamine (10 mM) (n=5), or palmitate (n=6) (50 µM). Continuous extracellular acidification (ECAR) (**A**) values are shown in response to substrate, Oligomycin A (1 µM) and Rotenone/AntimycinA (Rot/AA) (1 µM). Substrate induced ECAR (**B**) was calculated as the difference between baseline and acute substrate injection. Glycolytic reserve (**C**) was calculated as the difference in ECAR between baseline and Oligomycin A (p=0.0075). OciLy1 (n=6) (**D-F**) or BJAB (n=7) (**G-I**) WT and CD37KO cells were subjected to an acute injection of palmitate (50 µM) (**D**) or oleic acid (50 µM) (**G**), and oxygen consumption was monitored in response to Oligomycin A (1 µM), FCCP (1 µM) and Rotenone/AntimycinA (1 µM). Substrate induced OCR (**E, H**) was calculated as the difference between basal respiration and respiration after substrate injection (**H**; p=0.0221). Spare respiratory capacity (**F, I**) was calculated as the difference in OCR between baseline and FCCP (**F**; p=0.0386). ATP production (CPS) was determined for OciLy1 (**J, K**) WT and CD37KO cells that were supplemented with inhibitor of glycolysis, 2-DG (20 mM) (n=3, WT; p=0.0003) (**J**) or palmitate (n=6, KO; p=0.0130) (**K**). ATP production was quantified and plotted relative to non-treated BJAB CD53KO cells (n=3) supplemented with palmitate and glucose (**L**). Two-Way unpaired T-test (**B, C, E, F**) or Two-Way ANOVA with Tukey's Post-Hoc tests (**J-L**) were performed to check for significant differences between indicated groups, **p<0.01, ***p<0.001. Error bars represent mean +/- SD. Experiments were repeated twice yielding similar results. Source data are provided as a Source Data file.

Supplementary Fig. 2

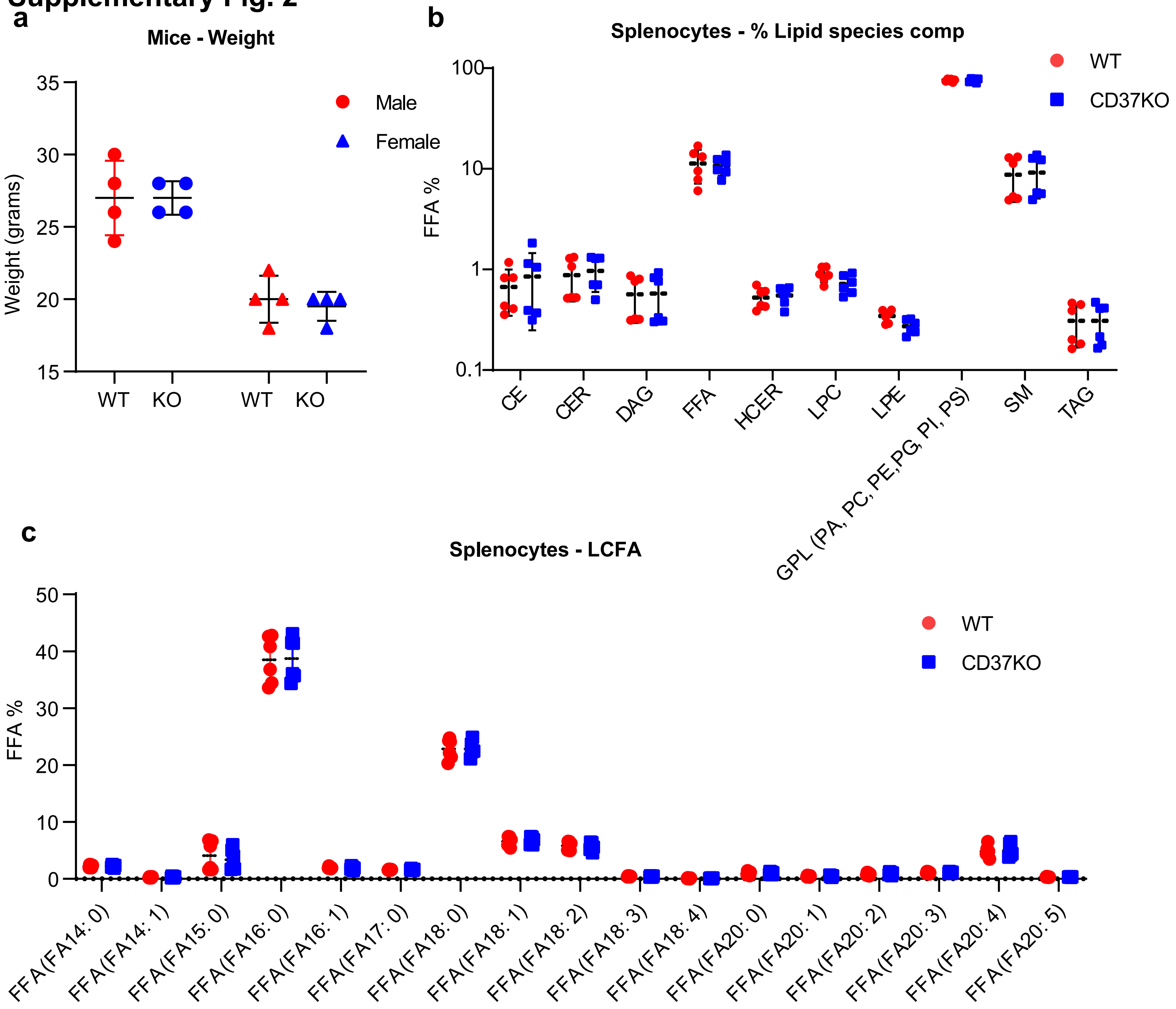

Figure S2. Intracellular lipid composition is not different between primary B cells from WT or CD37KO mice. Quantification of weight (grams) of male and female WT and CD37KO mice at comparable age (2 months) (n=4) (**A**). Composition of different lipid species (**B**) and long chain fatty acids (LCFA) (**C**) in freshly isolated CD43-, B220+ primary B cells of WT (n=6) or CD37KO (n=6) mice of 2-3 months old. Error bars represent mean +/- SD. Experiments were repeated twice yielding similar results. Source data are provided as a Source Data file.

Supplementary Fig. 3

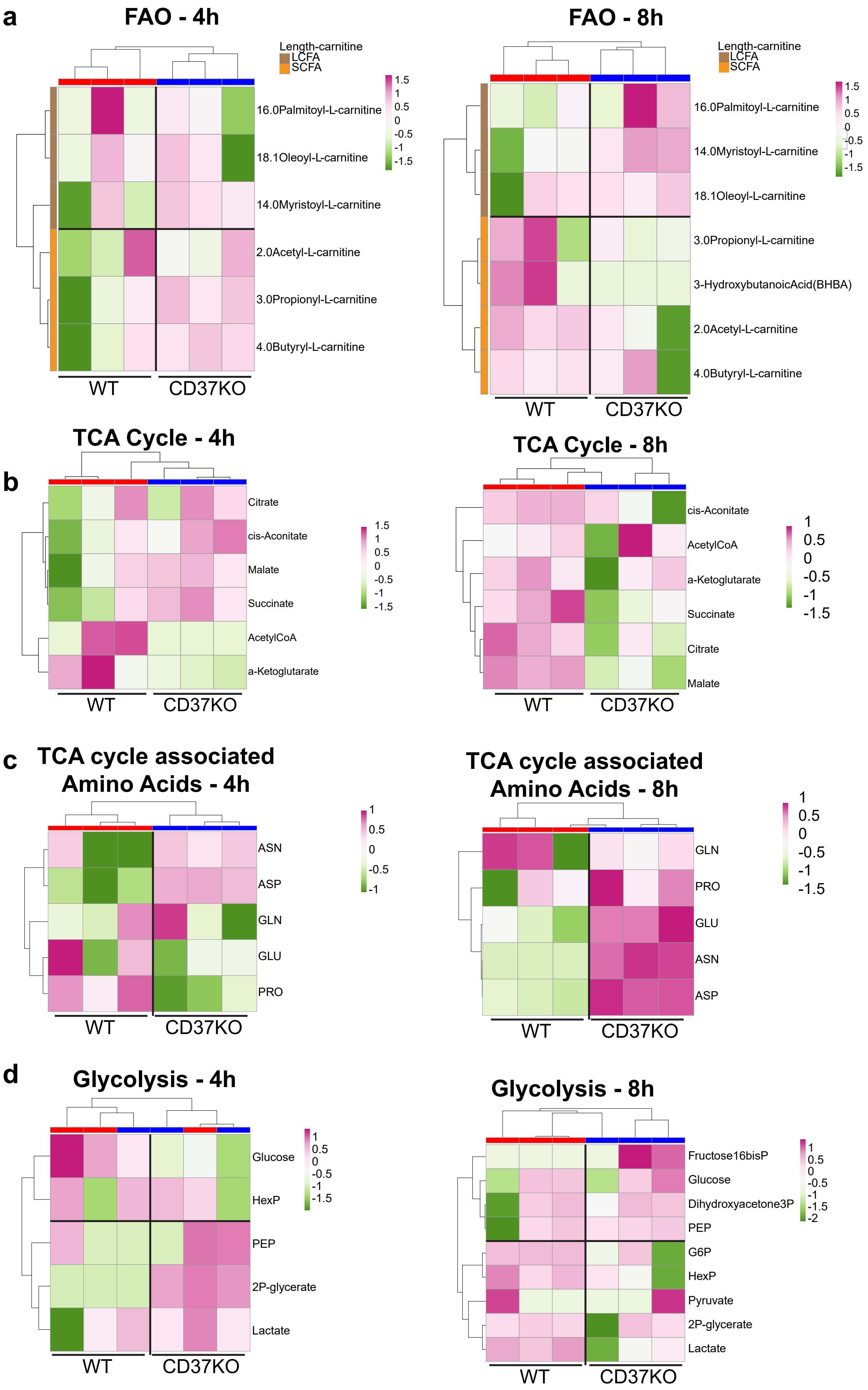

Supplementary Fig. 3

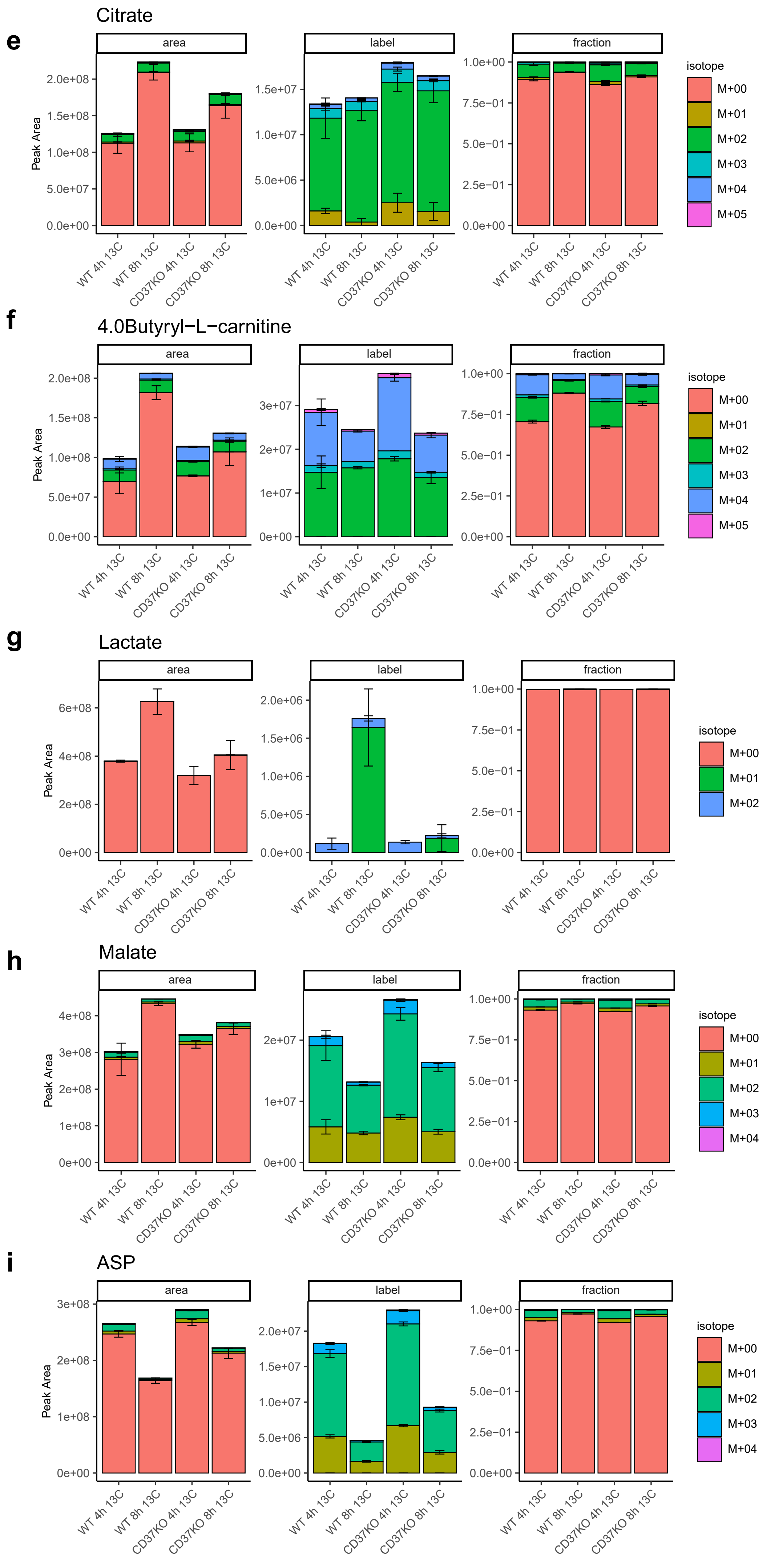

Figure S3. **^{13}C-incorporation in TCA intermediates and amino acids in CD37KO lymphoma cells**. WT (n=3 biologically independent samples per treated group) and CD37KO (n=3 biologically independent samples per treated group) lymphoma (BJAB) cells were supplemented with ^{13}C-palmitate (50 µM) for 4 and 8 hours. Total-isotope levels of ^{13}C-carnitines (**A**) are associated with fatty acid oxidation. ^{13}C-TCA-cycle intermediates (**B**) and TCA-cycle-derived ^{13}C-amino acids (**C**) and glycolysis ^{13}C-metabolites (**D**) after 4 or 8 hours of ^{13}C-palmitate supplementation were Log-transformed and analysed in greater detail with correlation-based-clustering. Peak area, label efficiency and label-fraction of relevant metabolites are shown (**E-I**). Error bars represent mean +/- SD. Experiments were performed once with biologically independent groups. Source data are provided as a Source Data file.

Supplementary Fig. 4

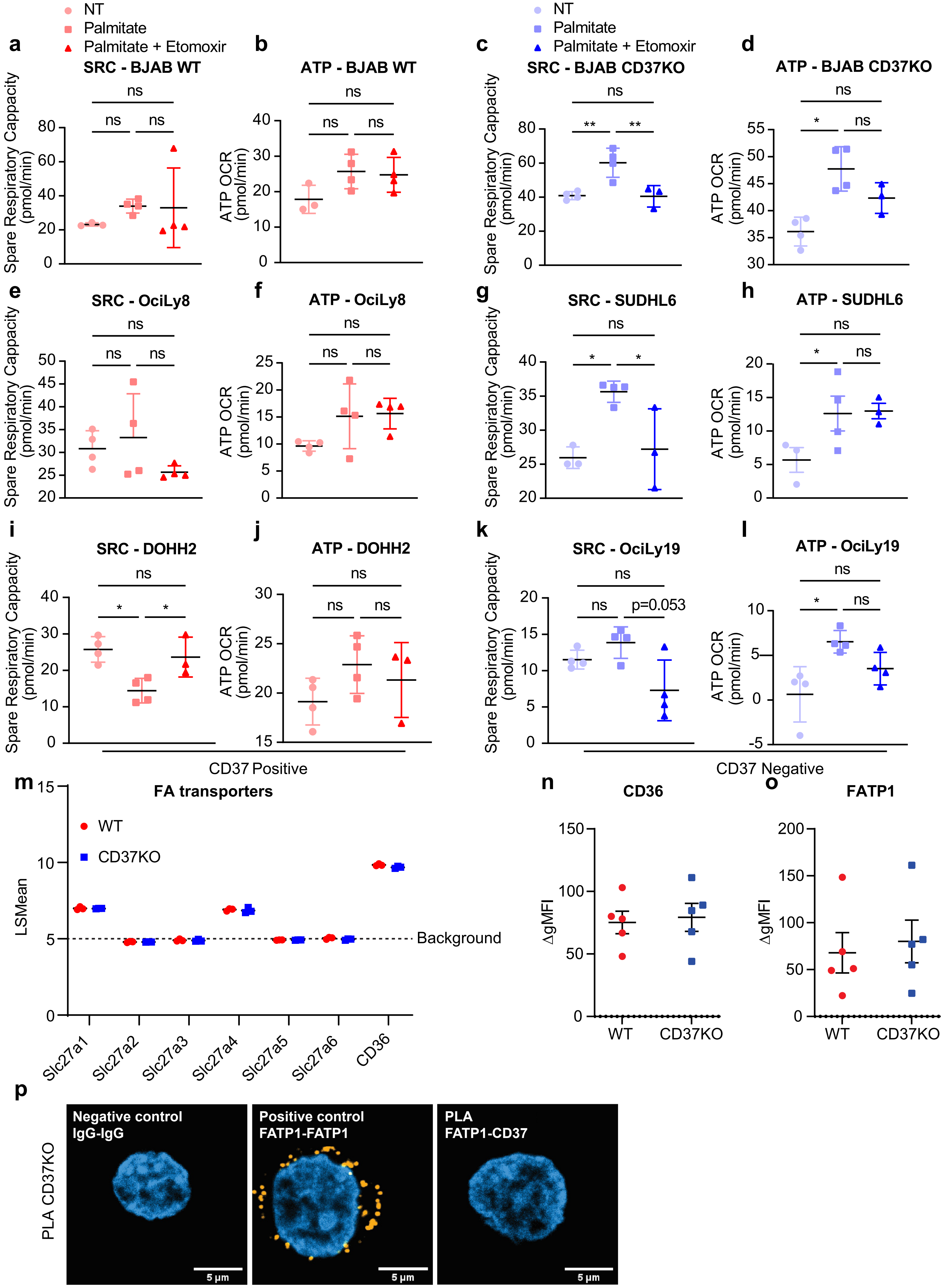

Figure S4. CD37-dependent fatty acid driven metabolic phenotype conserved in endogenous CD37 negative lymphoma cell lines and FA transporter expression on B cells. BJAB WT (n=4) (**A, B**) and CD37KO (n=4, SRC; p=0.0061, p=0.0084, ATP; p=0.0028) (**C, D**), CD37-positive OciLy8 (n=4) (**E, F**) and DOHH2 (n=4, SRC; p=0.0103, p=0.0412) (**I-J**) or CD37-negative SUDHL6 (n=4, SRC; p=0.0186, p=0.0347, ATP; p=0.0499) (**G-H**) and OciLy19 (n=4, ATP; p=0.0109) (**K-L**) lymphoma cells were subjected to an acute injection of palmitate (50 µM) with or without CPT1a inhibitor etomoxir (5 µM). Spare respiratory capacity was calculated as the difference in OCR between baseline and FCCP. ATP associated OCR was calculated as the difference in OCR between basal respiration and respiration after ATP-synthase inhibition with Oligomycin A. RNA quantification of FA transporters in 95% pure, CD43-, B220+ WT and CD37KO primary B cells (**M**). Data is displayed in Least-squares means (LSMeans). Data is from 3 CD37KO and 3 WT independent B cell isolations. Human WT and CD37KO lymphoma cells (BJAB) were stained for fatty acid transporters CD36 (n=5) (**N**) and FATP1 (n=5) (**O**) and analysed by flow cytometry. ΔgMFI=delta geometric Mean Fluorescence Intensity (gMFI target-gMFI isotype control). Visualization of in situ proximity ligation on CD37KO cells (BJAB) stained with isotype controls (**P**, left panel), positive control (two different primary antibodies against FATP1) (**P**, middle panel) and CD37 and FATP1 (**P**, right panel). Two-Way ANOVA with Tukey's Post-Hoc test (**A-L**) revealed significant differences between indicated groups, *p<0.05, **p<0.01. Error bars represent mean +/- SD. Experiments in **A-M** were performed once with biologically independent groups, experiments **N-P** were repeated at least three times yielding similar results. Source data are provided as a Source Data file.

Supplementary Fig. 5

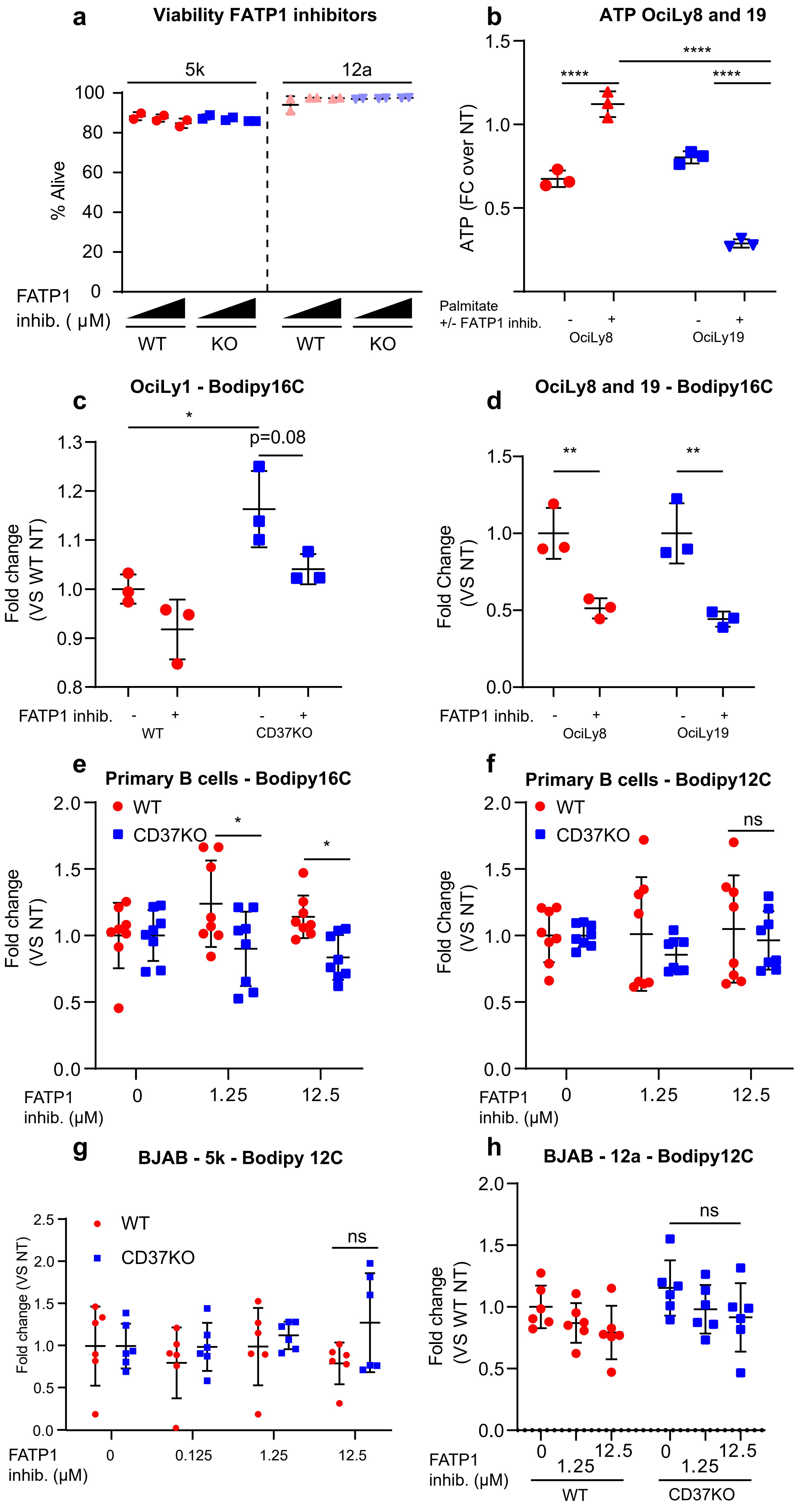

Figure S5. CD37-dependent metabolic switch is abolished in CD37KO human and mouse. Viability of BJAB WT (n=2) and CD37KO (n=2) lymphoma cells after 24 hours of incubation with 0, 1.25 µM or 12.5 µM FATP1 inhibitor 5k and 12a, determined with AnnexinV and PI staining (**A**). ATP quantification (CPS) in OciLy8 (n=3, p<0.0001) and OciLy19 (n=3, p<0.0001) (**B**) treated with palmitate and FATP1 inhibitor, compound 5k (12.5 µM). Uptake of palmitate analogue Bodipy FL C16 in OciLy1 WT (n=3) and CD37KO (n=3, WT- vs KO-; p=0.0249) (**C**), OciLy8 (n=3, p=0.0044) or OciLy19 (n=3, p=0.0019) (**D**) and primary B cells (n=8, 1.25 µM; p=0.0193, 12.5 µM; 0.0398) (**E**) as a result of palmitate and FATP1 inhibitor treatment. Uptake of palmitate analogue Bodipy FL C12 in primary B cells (**F**) in response to FATP1 inhibitor (compound 5k) treatment. Uptake in BJAB WT (n=6) and CD37KO (n=6) cells of Bodipy FL C12 Bodipy FL C12 as a result of FATP1 inhibition with inhibitor compound 5k (**G**) and 12a (**H**) (12.5 µM). Two-Way ANOVA with Tukey's Post-Hoc test (**A-H**) revealed significant differences between indicated groups, *p<0.05, **p<0.01, ***p<0.001, ****p<0,0001. Error bars represent mean +/- SD. Experiments were repeated twice yielding similar results. Source data are provided as a Source Data file.

Supplementary Fig. 6

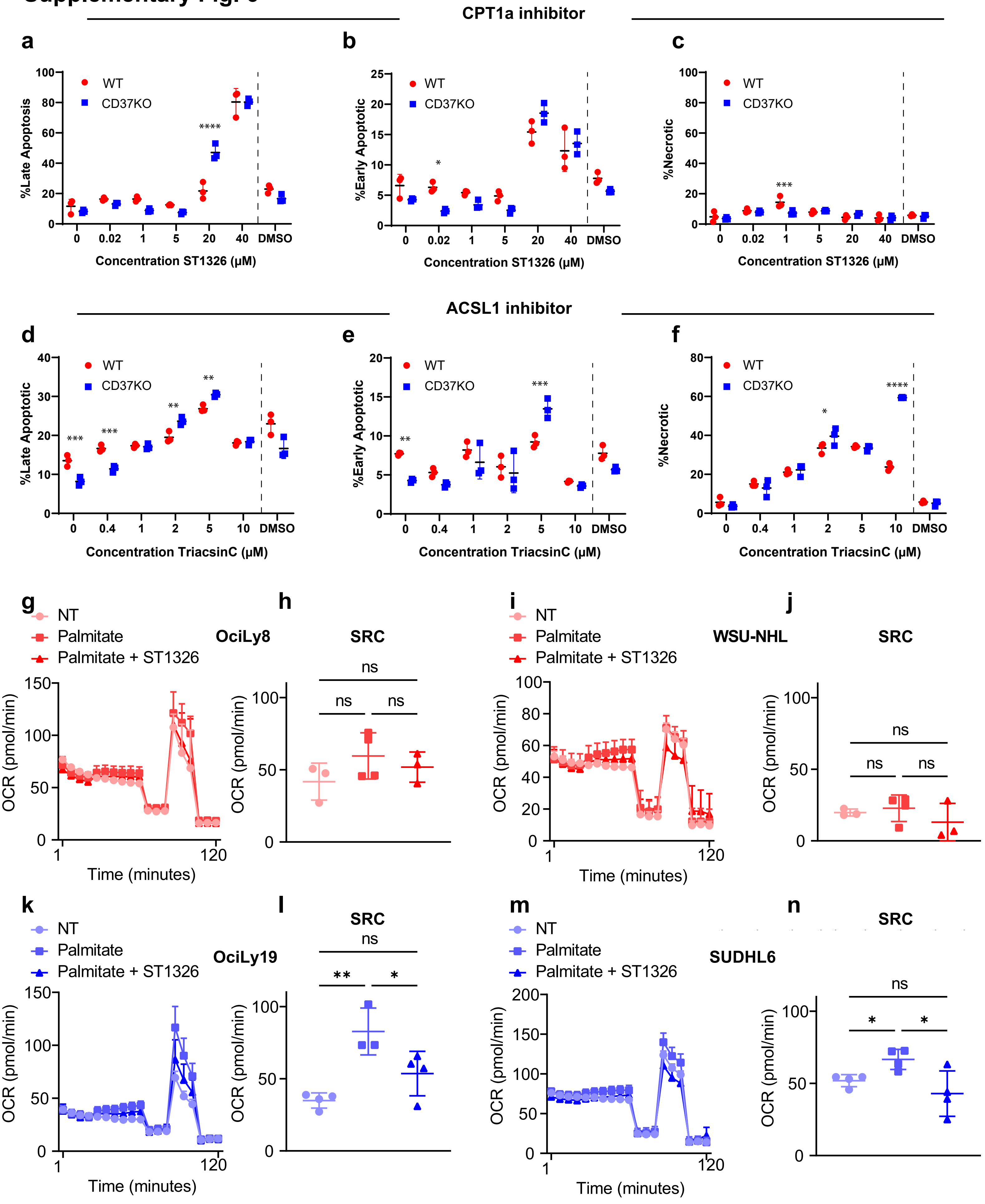

Figure S6. CD37KO and CD37-negative lymphoma cells are susceptible to CPT1a inhibition. Effects of 24 hours CPT1a inhibition with ST1326 at indicated concentrations (n=3 per concentration) on late apoptosis (**A:** 20 µM; p<0.0001), early apoptosis (**B:** 0.02 µM; p=0.0152) or necrosis (**C**: 1 µM; p=0.0003). Effects of 24 hours ACSL1 inhibition with TriacsinC at indicated concentrations on late apoptosis (**D**: 0 µM; p=0.0002, 0.4 µM; p=0.0002, 2 µM; p=0.0024, 5 µM; p=0.0073), early apoptosis (**E:** 0 µM; p=0.0048, 5 µM; p=0.0006) or necrosis (**F**: 2 µM; p=0.0195, 10 µM; p<0.0001). CD37-positive (OciLy8, n=4 (**G**) and WSU-NHL, n=4 (**H**)) and CD37-negative (OciLy19, n=4 (**K**), and SUDHL6, n=4 (**M**)) B cell lymphomas were cultured in nutrient-restricted medium and stimulated with palmitate (50 µM) either with or without CPT1a inhibitor ST1326 (5 µM). Continued oxygen consumption was monitored in response to Oligomycin A (1 µM), FCCP (1 µM) and Rotenone/AntimycinA (1 µM). Spare respiratory capacity (**H, J, L, N**) was calculated as the difference in OCR between baseline and FCCP (**L**: p=0.0024, p=0.0299, **N**: p=0.0111, p=0.0339). Two-Way ANOVA with Tukey's Post-Hoc test (**A-N**) were performed to check for significant differences between indicated groups, *p<0.05, **p<0.01, ***p<0.001, ****p<0,0001. Error bars represent mean +/- SD. Experiments **A-F** were repeated twice yielding similar results, experiments **G-N** were repeated once. Source data are provided as a Source Data file.

Supplementary Fig. 7

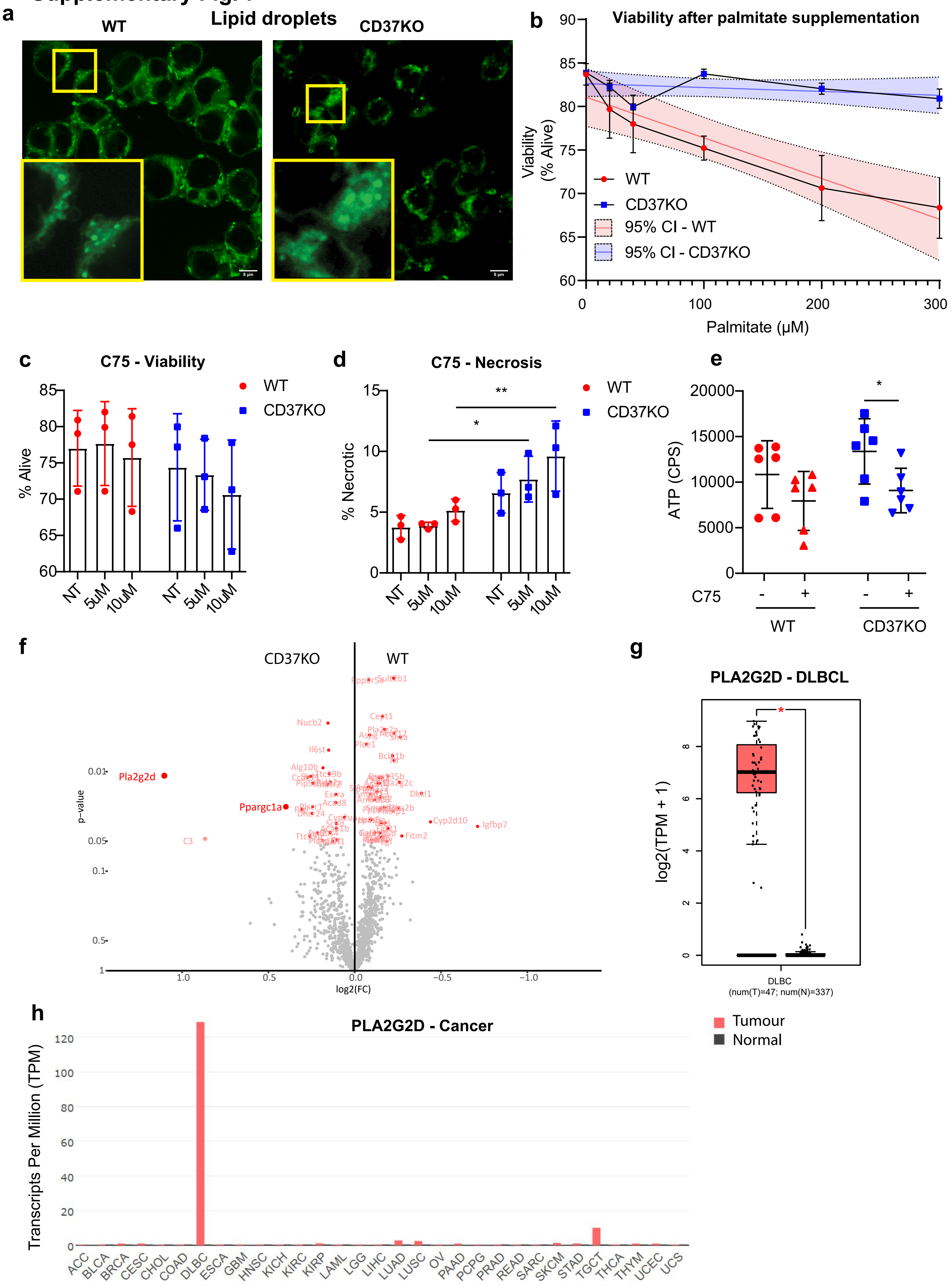

Figure S7. Lipid droplet visualisation and FA synthase inhibition in CD37KO B cell lymphoma and database analysis of CD37KO-associated phospholipase. Human WT and CD37KO B-cell lymphomas were stained with Bodipy 493/503 that accumulates in neutral lipid regions found in lipid droplets and visualized with confocal microscopy (**A**). Scale bar is 5 μm. Viability was measured after 48 hours incubation with increasing concentrations of palmitate (**B**). Blue (squares) and red (circles) zones (**B**) represent the 95% Confidence interval for the trend. Viability (**C**) and necrosis (**D**) were assessed for WT (n=3) and CD37KO (n=3) lymphoma B cells (BJAB) in response to 24 hours of fatty acid synthase (FAS) inhibition with C75 (5 μM; p=0.0158, 10 μM; p=0.0065). ATP quantification (CPS) in WT (n=6) and CD37KO (n=6, p=0.0356) lymphoma B cells (BJAB) after 24 hours of fatty acid synthase (FAS) inhibition with C75 (10 μM) (**E**). Quantification of RNA expression of fatty acid metabolism-associated genes in primary CD43-, B220+ B cells of 3 WT and 3 CD37KO mice (**F**). Quantification of expression found in database (Gene Expression Profiling Interactive Analysis, GEPIA) analysis for PLA2g2D in Diffuse Large B cell lymphoma; Median (Tumour, N=47): 128.791, Min/Max: 3/500; Median (Normal, N=337): 0.010, Min/Max: 0/1; Box displays upper and lower quartile, Log2(Fold Change): 7.006 (**G**) and all other available cancers in the database (**H**). Two-Way ANOVA with Tukey's Post-Hoc tests (**C-E**) were performed to check for significant differences between indicated groups, *p<0.05, **p<0.01. Error bars represent mean +/- SD. Experiments **A-E** were performed three times yielding similar results. Source data are provided as a Source Data file.

Gating Strategy

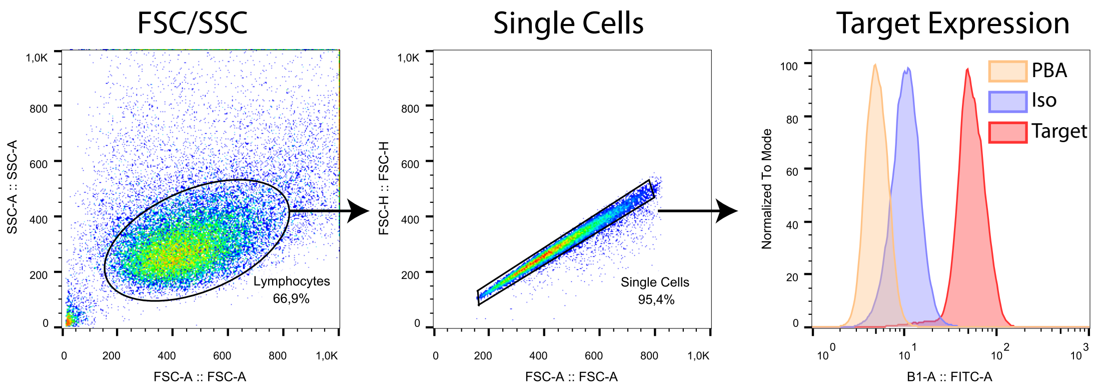

Supplement: Supplementary file 1 — Supplementary Information [file 41467_2022_33138_MOESM1_ESM.pdf]
